# A case of lepromatous leprosy in Arizona, United States

**DOI:** 10.1016/j.jdcr.2023.10.021

**Published:** 2023-11-04

**Authors:** Katherine K. Robbins, Lucía A. Luna-Wong, Matthew Adams, Frances I. Ramos-Herberth

**Affiliations:** a Division of Dermatology, University of Arizona College of Medicine – Tucson, Tucson, Arizona; b Department of Medicine, University of Arizona College of Medicine – Tucson, Tucson, Arizona; c Department of Medicine, Southern Arizona VA Health Care System, Tucson, Arizona; d Department of Dermatology, University of Connecticut School of Medicine, Farmington, Connecticut; e Department of Medicine, VA Connecticut Health Care System, Newington, Connecticut

*To the Editor:* We read with interest the case series by Domozych et al^1^ and wish to share our case of a patient diagnosed with lepromatous leprosy in Arizona who lived extensively in Florida and denied direct armadillo exposure.

A 79-year-old Caucasian man with a history of well-controlled type 2 diabetes mellitus, presented with a 10-year history of a progressive eruption of pink patches on the trunk and extremities. Two years before presentation, he began to develop hypoesthesia of the affected patches, peripheral neuropathy of the distal lower extremities, and marked swelling of bilateral hands and feet. He reported difficulty ambulating, which he attributed to his neuropathy, and described hyperalgesia of fingertips that prevented him from performing daily activities, such as tying shoelaces and buttoning his shirts.

On examination, he had symmetrically distributed ill-defined pink to violaceous patches with fine scale affecting the face, trunk, and extremities affecting 70% of his total body surface area (Fig 1, *A*). He also had significant swelling of the hands and feet (Fig 1, *B*). Numerous patches had loss of light touch sensation.

**Fig 1:**
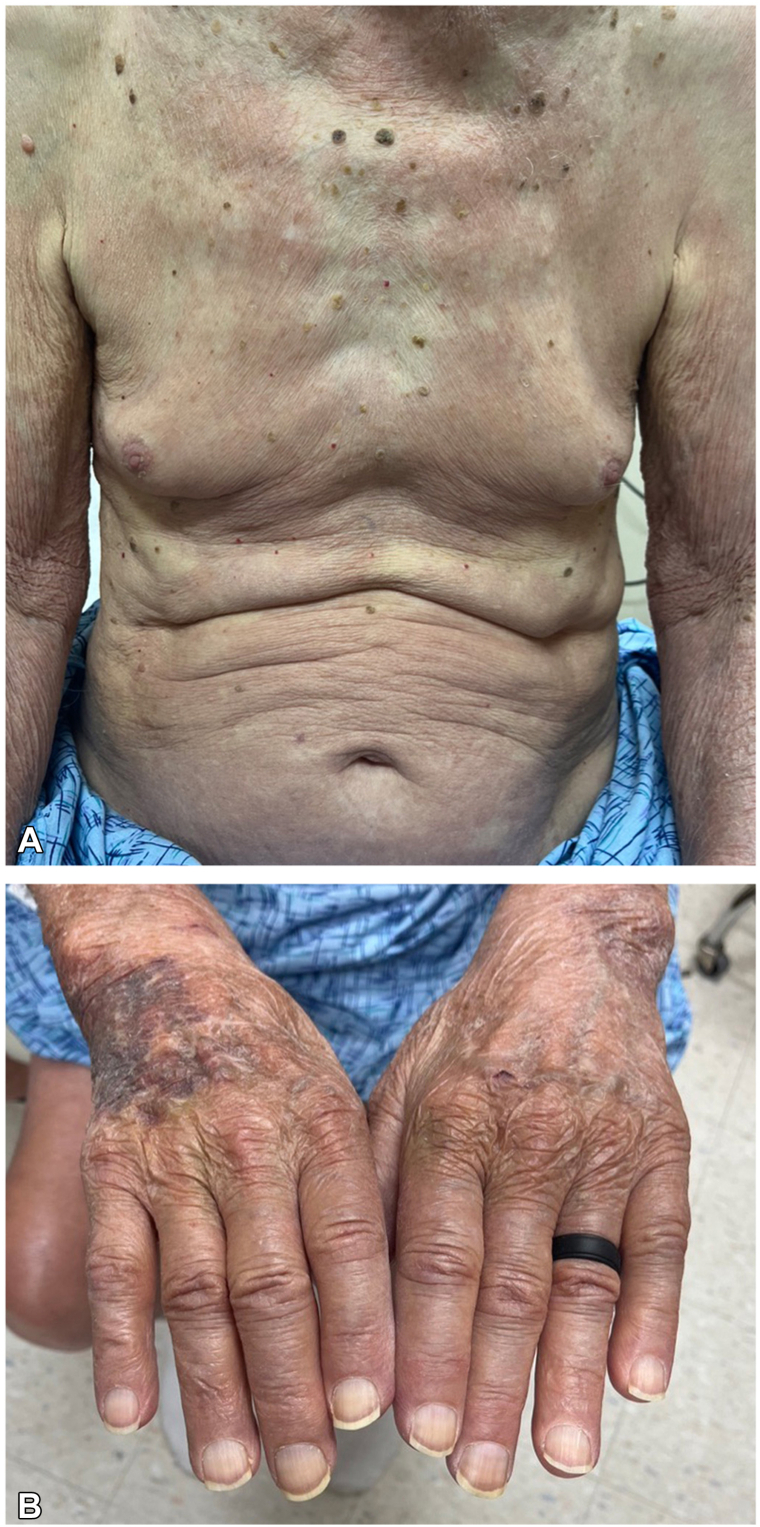
Lepromatous leprosy. **A**, Numerous symmetric, ill-defined pink patches on the trunk and upper extremities. **B**, Swelling of the digits.

Punch biopsy of 1 abdominal lesion demonstrated perivascular and perineural lymphohistiocytic infiltrate containing foamy histiocytes extending into the reticular dermis and involving cutaneous nerves (Fig 2, *A* to *C*). Fite staining revealed numerous acid-fast bacilli within nerves and histiocytes (Fig 2, *D*). A tissue sample sent for polymerase chain reaction at the National Hansen’s Disease Program identified *Mycobacterium leprae* as the culprit species. A diagnosis of lepromatous leprosy was made due to the widespread cutaneous involvement and the number of bacilli seen on histology.^2^

**Fig 2:**
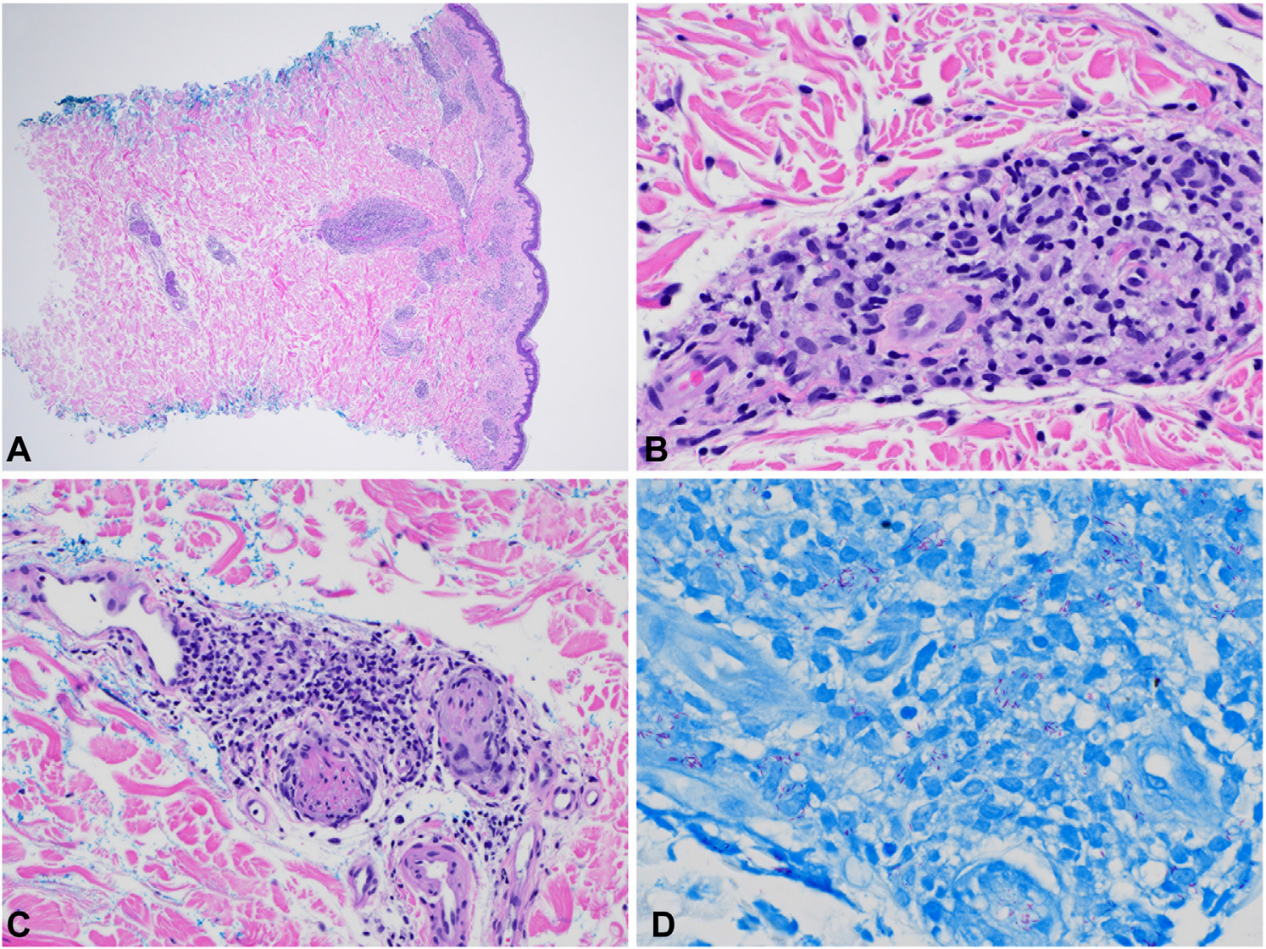
Leprosy. **A**, Mixed lymphohistiocytic infiltrate extending into the reticular dermis. **B**, Mixed chronic-histiocytic inflammation. **C**, Inflamed cutaneous nerve. **D**, Numerous acid-fast bacilli. (**A**, **B**, and **C**, Hematoxylin-eosin stain; original magnifications: A, '20; B, '400; and C, '200.) (**D**, Fite-stain; original magnification: '600.)

To decrease the risk of type 1 reversal reaction, erythema nodosum leprosum, and Lucio phenomenon, a prednisone taper, weekly methotrexate 15 mg, and folate supplementation were initiated before starting monthly rifapentine 600 mg, moxifloxacin 400 mg, and minocycline 100 mg.^3^ He is tolerating therapy well and notes improvement in his daily life, including the ability to dress himself once again.

The patient lived in Florida for 50 years where he worked as a landscaper for 25 years before moving to the southwest in 2015. Travel history included repeated mission trips to Chihuahua, Mexico and vacations to the Caribbean in the 5 to 10 years leading up to diagnosis, which have lower leprosy rates compared with Florida, where Hansen disease is now considered endemic.^4^^,^^5^ Although he denied exposures to a person with leprosy or to 9-banded armadillos, the main zoonotic reservoir in the United States, *M. leprae* has been isolated from the soil of endemic regions.^2^^,^^4^ Similar to other autochthonous cases recently reported in Florida, our patient’s occupation in landscaping suggests the possibility of leprosy transmission from indirect exposure to land inhabited by the 9-banded armadillo.^1^^,^^5^

This report demonstrates the need for a high index of suspicion for leprosy when seeing patients with refractory rashes and neuropathy (eg, hypoesthesia and hyperalgesia), and who have spent time not only in typical leprosy endemic areas, such as South America, Asia, or Africa, but also in new areas of concern for autochthonous leprosy, such as central Florida.^1^^,^^5^

## Conflicts of interest

None disclosed.

## References

[1] Domozych R., Kim E., Hart S., Greenwald J. (2016). Increasing incidence of leprosy and transmission from armadillos in Central Florida: a case series. JAAD Case Rep.

[2] Maymone M.B.C., Laughter M., Venkatesh S. (2020). Leprosy: clinical aspects and diagnostic techniques. J Am Acad Dermatol.

[3] Maymone M.B.C., Venkatesh S., Laughter M. (2020). Leprosy: treatment and management of complications. J Am Acad Dermatol.

[4] Leprosy D.K., Bennett J.E., Dolin R., Blaser M.J. (2019). Mandell, Douglas, and Bennett’s Principles and Practice of Infectious Diseases.

[5] Bhukhan A., Dunn C., Nathoo R. (2023). Case report of leprosy in Central Florida, USA, 2022. Emerg Infect Dis.

